# The effects of selected flavonoids on cytochromes P450 in rat liver and small intestine

**DOI:** 10.2478/v10102-009-0018-y

**Published:** 2009-09-28

**Authors:** Jitka Křížková, Kamila Burdová, Marie Stiborová, Vladimír Křen, Petr Hodek

**Affiliations:** 1 Department of Biochemistry, Faculty of Science, Charles University in Prague, Hlavova 2030, 128 40, Prague 2, Czech Republic; 2 Institute of Microbiology, Center for Biocatalysis and Biotransformation, Academy of Sciences of the Czech Republic, Vídeňská 1083, 142 20, Prague 4, Czech Republic

**Keywords:** flavonoids, cytochrome P450, small intestine, liver, induction

## Abstract

In recent years, the consumption and use of dietary supplements containing concentrated phytochemicals (e.g. flavonoids) increased dramatically. Flavonoids, as foreign compounds (xenobiotics), have great potential to modulate the activity of cytochrome P450s (CYPs), xenobiotic-metabolizing enzymes involved in the activation and detoxification of food and environmental carcinogens. Thus, the aim of this study was to investigate the effects of model glycosylated and deglycosylated flavonoids on CYPs in rat liver and small intestine, as the two main organs responsible for xenobiotic metabolism, after *p.o.* administration by gastric gavages. The effects of two glycosylated flavonoids (isoquercitrin, rutin) and their aglycone (quercetin) on CYPs were determined using Western blotting technique and specific activity assays with alkyl-resorufin derivatives. In liver microsomes, a considerable increase of all the measured marker activities (EROD, MROD, PROD) was observed only after isoquercitrin treatment. To evaluate the effects of flavonoids on CYPs along small intestine, the tissue was dissected into proximal (near pylorus), middle and distal parts. Of all the tested compounds, isoquercitrin was the most efficient CYP inducer, namely in the middle part of small intestine. Obtained data demonstrate the different effects of flavonoid glycosides and aglycone on CYP expression in rat liver and small intestine. Since these phytochemicals are xenobiotics, and thus they can increase the human risk of cancer development, their consumption in large quantities should be carefully considered.

## Introduction

According to the World Health Organization statistics, cancer is one of the leading causes of death in the human population worldwide for more than 50 years. Moreover, colorectal and gastrointestinal tract cancer are one of the main types of cancer leading to overall cancer mortality. Prevention consisting in a healthy lifestyle and a natural diet is suggested to be one of the main approaches to reduce cancer risk. In recent years, the consumption and use of dietary supplements containing concentrated phytochemicals increased dramatically. Flavonoids present in foods (fruits, vegetables, herbs, beverages) and dietary supplements have the greatest potential to modulate activity of xenobiotic-metabolizing enzymes (Hodek *et al*., 2002). Among proteins interacting with flavonoids, cytochrome P450s (CYPs), monooxygenases metabolizing xenobiotics (e.g. drugs, carcinogens), play the most prominent role. Since phytochemicals are foreign compounds (xenobiotics), their consumption in large quantities should be carefully considered. Further, potential dietary supplement-drug and food-drug interactions, leading to an overdose or the loss of the drug therapeutic effects, have been reported (Jang *et al*., 2004; Morris and Zhang, 2006). Beside these interactions, the negative properties of these dietary supplements may have their origin in: (i) their own toxicity, (ii) metabolic conversion into cytotoxic or mutagenic agents, (iii) induction of carcinogen activating enzymes, and (iv) effect on human microflora (Hodek *et al*., 2009).

Biological benefits of flavonoids depend on their bioavailability after oral ingestion. In general, flavonoids are metabolized mainly in liver and intestines. Natural flavonoids usually occur as β-glycosides that are relatively hydrophilic and thus do not diffuse passively across biological membranes. Up to now, various studies have been carried out to obtain pharmacological data in order to elucidate the absorption, metabolism and disposition of flavonoids. However, some of the results are controversial, and the complexity of the delivery process from the administration site to the target organs, possibly via receptors, makes generalization of flavonoid metabolism hardly possible. It has been suggested, that glycoside transporters (Gee *et al*., 1998), β-glucosidases (Day *et al*., 1998), colon microflora (Parkar *et al*., 2008, Atkinson *et al*., 2005) and conjugation of aglycones to glucuronides are the main metabolic pathways of flavonoids in small intestine and liver (Zhang *et al*., 2007).

The aim of this study is to examine the effects of glycosylated flavonoids, isoquercitrin and rutin, on cytochrome P450 expression in comparison with their aglycone, quercetin, in rat liver and small intestine, as the two main organs responsible for xenobiotic metabolism.

## Material and Methods

### Chemicals

β-Naphthoflavone, quercetin dihydrate, rutin hydrate (quercetin-3-rutinoside), NADPH, bicinchoninic acid, resorufin, 7-ethoxyresorufin, 7-methoxyresorufin, 7-pentoxyresorufin, and anti-chicken IgG alkaline phosphatase conjugate, BCIP/NBT tablets were purchased from Sigma Chemical Co., (St. Louis, MO). Isoquercitrin (quercetin-3-β-D-glucoside, purity 98.2%) was prepared by Dr. Křen. Specific chicken anti-CYP1A1/2 and anti-CYP2B1/2 antibodies were prepared by Dr. Hodek. All other chemicals were purchased from standard commercial sources and were of the highest quality available.

### Animal treatment and preparation of microsomes

All studies with rats were carried out in accordance with guidelines of the Ethical Committee of Faculty of Science. Male Wistar rats (150 g) obtained from AnLab, Czech Republic, were housed in groups of 3 in wire cages at 22 °C with a 12 h light/dark period and *ad libitum* diet (ST-1 diet from Velaz, Czech Republic) and water access. The tested chemopreventive compounds (60 mg/kg body weight) were administered *p.o.* by gastric gavages, dissolved in sunflower oil (1 ml), daily for 5 consecutive days. The control group was treated with 1 ml of sunflower oil only. The treated rats were fasted overnight and, twenty-four hours after the last treatment, they were sacrificed. Microsomes were prepared from sections of small intestine and whole liver, immediately after sacrificing the rats, as described previously (Křížková *et al*., 2008). Tissues from 3 rats were pooled for each microsomal preparation. Small intestine was removed circa 2 cm under the stomach, divided into three parts (proximal near pylorus, middle, distal), each approximately 20 cm long. Microsomes from animals pretreated with rutin were available only from proximal and distal parts. Microsomal fractions were stored at −80 °C before use.

### Determination of protein concentration and Western blot analysis

Protein concentration in microsomes was measured according to Smith *et al*. (1985) using bicinchoninic acid with bovine serum albumin as the standard. The CYP1A and CYP2B were detected by Western blotting on Immobilon-P membrane (Millipore, Bedford, MA) using specific chicken anti-CYP1A1/2 and anti-CYP2B1/2 antibodies. Since the content of cytochrome P450s in small intestine is lower compared to liver microsomes, the samples used for SDS-electrophoresis (30 µg protein/well, 8% polyacrylamide gel) and following Western blotting analysis were twice as concentrated as the ones from liver (15 µg protein/well). Visualization was performed using an anti-chicken IgG alkaline phosphatase-conjugated antibody and BCIP/NBT tablets containing 10 mg substrate for alkaline phosphatase.

### Enzyme assays

The CYP activity of microsomes was characterized by use of alkyl-resorufin derivatives. 7-Ethoxyresorufin-*O*-deethylase (EROD), 7-methoxyresorufin-*O*-demethylase (MROD) and 7-pentoxyresorufin-*O*-depentylase (PROD) activity assays were determined according to the method described by Burke and Mayer (1974). Formation of the resorufin was continuously measured for 10 minutes at room temperature by monitoring its fluorescence (excitation and emission wavelengths of 530 and 585 nm, respectively). The dealkylation rate was estimated on the basis of a resorufin standard curve.

## Results

### Effects of flavonoids on CYP enzyme activities in liver and small intestine

**Figure 1:**
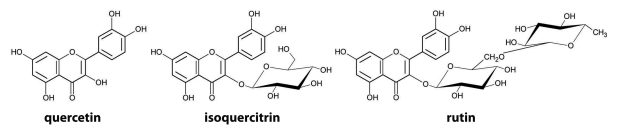
Structure of tested flavonoids.

**Figure 2:**
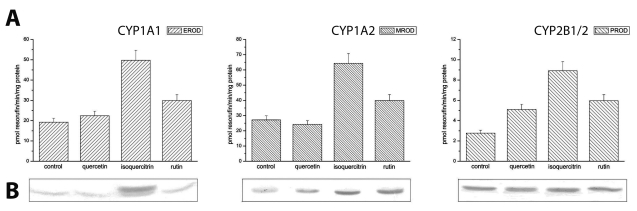
**(A)** Effects of flavonoids on EROD, MROD and PROD activity of CYP1A1, CYP1A2 and CYP2B1/2, respectively, in rat liver microsomes after dietary exposure to flavonoids (60 mg/kg body weight) for 5 days. Bars represent the means±SD of 3 determinations. **(B)** Immunodetection of CYP1A1, CYP1A2 and CYP2B1/2 in liver microsomes. Electrophoresed microsomal proteins (15 µg) were transferred to Immobilon-P membrane and probed with antibody against CYP1A1, CYP1A2 and CYP2B1/2.

Two glycosylated flavonoids (isoquercitrin, rutin), and their aglycone, quercetin (Figure 1), were tested for their effects on CYP activities in liver and small intestine after *p.o.* administration. In respect of liver microsomes, Figure 2A illustrates EROD, MROD and PROD activities for CYP1A1, CYP1A2 and CYP2B1/2, respectively. In all cases, isoquercitrin increased significantly CYP activities, with the highest increase being in PROD activity, more than 3-times compared to untreated rats. Rutin administration also enhanced all measured specific activities, in the same manner but to a lesser extent than isoquercitrin.

**Figure 3:**
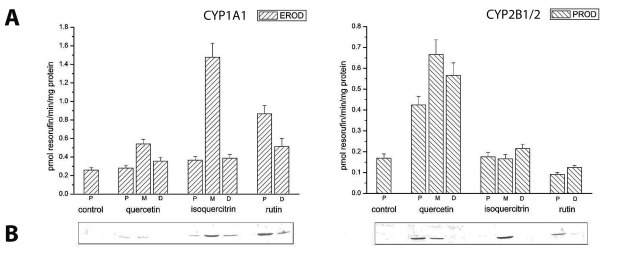
**(A)** Effects of flavonoids on EROD and PROD activity of CYP1A1 and CYP2B1/2, respectively, in the proximal (P), middle (M) and distal (D) part of rat small intestine after dietary exposure to flavonoids (60 mg/kg body weight) for 5 days. Bars represent the means±SD of 3 determinations. **(B)** Immunodetection of CYP1A1 and CYP2B1/2 in the proximal (P), middle (M) and distal (D) part of rat small intestine. Electrophoresed microsomal proteins (30 µg) were transferred to Immobilon-P membrane and probed with antibody against CYP1A1 and CYP2B1/2.

For comparison between two organs highly exposed to xenobiotics (liver and small intestine), the effects of flavonoids on CYPs in small intestine dissected into three parts were also studied. In small intestine, an identical trend of quercetin and isoquercitrin effects on CYP activities was observed (Figure 3A). Surprisingly, the highest increase of EROD and PROD activity was determined in the middle part of the small intestine. All the tested compounds did not affect MROD activity compared to untreated rats (data not shown). Results from the administration of a synthetic flavonoid, β-naphthoflavone (data not shown), showed the same trend as in case of rutin treatment.

### Western blot analysis of CYPs expression in small intestine and liver

To confirm data from CYP marker activities, we examined the levels of CYPs protein using Western blotting technique. In respect of liver microsomes, the results of Western blot detection of CYPs are shown in Figure 2B. A strong induction of CYP1A1 was observed only after isoquercitrin treatment, which along with rutin also induced CYP1A2. On the other hand, a considerable increase in CY2B1/2 expression at the protein level was not demonstrated.

Although CYPs in extrahepatic tissues are several times less abundant than in liver, we succeeded in detecting CYP1A1 and CYP2B1 in small intestine (Figure 3B). Isoquercitrin caused strong induction of both detected CYPs, CYP1A1 and CYP2B1, in the middle part of small intestine. However, in the proximal part, a marked induction of CYP2B1 was detected in rats pretreated with quercetin and rutin, which further induced CYP1A1.

## Discussion

Cancer remains one of the leading causes of death in the human population worldwide. The new trend of healthy lifestyle together with an increasing consumption of dietary supplements containing *i.e.* flavonoids evoke concerns regarding their unlimited consumption, as their side effects are not sufficiently known. Chemopreventive compounds are added to dietary supplements mainly for their potential health-beneficial activities. Flavonoids have the potential to modulate the activity of cytochromes P450, mainly CYP1A subfamily. Unfortunately, CYP1A1 and CYP1A2 are involved in the activation of food and environmental carcinogens. Thus, the intake of CYP1A inducers might increase the human risk of cancer development.

The gastrointestinal tract is the site exposed to exogenous compounds of food or orally delivered drugs, therefore, the present study is focused on the effects of flavonoids on CYPs in the two organs highly exposed to xenobiotics, liver and small intestine of rat model organism.

To simulate real human intake of flavonoids, rats were treated *p.o.* by gastric gavages with the tested compounds for 5 consecutive days. In this study, we demonstrated that tested flavonoids, as one of the main components of dietary supplements, induced procarcinogen activating enzymes, CYP1A/2 and CYP2B1/2, in liver and small intestine.

In rat liver, based on determined protein level and specific enzyme actvities, isoquercitrin was the most efficient inducer of CYP1A1 and CYP1A2. On the other hand, aglycone quercetin had no effect either on the protein level or on EROD, which is in accordance with the results from Canivenc *et al*. (1996). Contrary to that, it was observed that quercetin caused a concentration-dependent increase of CYP1A1 mRNA in MCF7 cells (Ciolino *et al*., 1999). A study with commercial herbal supplement FastOne™ showed an increase of both EROD activity and protein level of CYP1A2, and thus suggests that intake of the herbal supplement may increase the risk of colorectal cancer in humans with the rapid NAT2 phenotype (Ryu and Chung, 2003).

The same induction pattern as in liver was observed in small intestine; the highest increase of EROD measured in the middle part of small intestine and protein level of CYP1A1 was caused by isoquercitrin. On the contrary, quercetin increased PROD activity in all three parts of small intestine and induced CYP2B1 in proximal and middle parts. All the tested compounds caused an increase of PROD, compared to untreated rats, in the distal part of small intestine but CYP2B1/2 was not detected, thus it seems necessary to further investigate the effects of flavonoids on CYPs in small intestine.

The different effects of tested flavonoids on CYP expression and activities in small inestine and liver could be explained by the diverse bioavailability of particular flavonoids, which involve rate of transport to lumen, conjugation or further metabolism. Moreover, different inductive effects of flavonoid glycosides (isoquercitrin, rutin) and aglycone (quercetin) confirmed the complexity of the delivery process from the administration site to the target organs.

In conclusion, we have demonstrated different effects of flavonoids on xenobiotic-metabolizing enzymes in rat liver and small intestine. Therefore, this study indicates that the consumption of dietary supplements containing flavonoids without any limitation should be carefully considered. Moreover, we confirmed that the absorption and metabolism of flavonoids depend on the presence and type of sugar linked to aglycone.
